# Antigenicity of key hepatitis C virus E1E2 glycoprotein neutralizing sites is genotype independent

**DOI:** 10.1099/jgv.0.002201

**Published:** 2026-01-13

**Authors:** Jessica L. Mimms, Ariadne Sinnis-Bourozikas, Nathaniel R. Felbinger, Nicole Frumento, Harry T. Paul, Arvind H. Patel, Zhenyong Keck, Steven K. H. Foung, Mansun Law, Richard A. Urbanowicz, Alexander W. Tarr, Jonathan K. Ball, Brian G. Pierce, Justin R. Bailey

**Affiliations:** 1 Division of Infectious Diseases, Department of Medicine, Johns Hopkins University School of Medicine, Baltimore, MD, USA; 2 University of Maryland Institute for Bioscience and Biotechnology Research, Rockville, MD, USA; 3 Department of Cell Biology and Molecular Genetics, University of Maryland, College Park, MD, USA; 4 Peter Medawar Building for Pathogen Research, Nuffield Department of Clinical Medicine, University of Oxford, Oxford, UK.; 5 MRC–University of Glasgow Centre for Virus Research, Glasgow, UK; 6 CVR-CRUSH, MRC-University of Glasgow Centre for Virus Research, Glasgow, UK; 7 Department of Pathology, Stanford University School of Medicine, Stanford, CA, USA; 8 Department of Immunology and Microbiology, The Scripps Research Institute, La Jolla, CA, USA; 9 Department of Infection Biology and Microbiomes, Institute of Infection, Veterinary and Ecological Sciences, University of Liverpool, Liverpool, UK; 10 School of Life Sciences, Faculty of Medicine and Health Sciences, The University of Nottingham, Nottingham, UK; 11 Wolfson Centre for Global Virus Research, The University of Nottingham, Nottingham, UK; 12 NIHR Nottingham Biomedical Research Centre, Nottingham University Hospitals NHS Trust, Nottingham, UK; 13 Liverpool School of Tropical Medicine, Liverpool, UK; 14 Department of Molecular Microbiology and Immunology, Johns Hopkins University Bloomberg School of Public Health, Baltimore, MD, USA

**Keywords:** hepatitis C virus (HCV), hepatitis C virus (HCV) vaccine, hepatitis C virus pseudoparticles (HCVpp), neutralizing antibody, neutralizing breadth

## Abstract

The development of an effective prophylactic hepatitis C virus (HCV) vaccine is a priority to achieve global elimination of the virus. Accurate assessment of the neutralizing breadth of antibodies induced by vaccines and a clear understanding of the antigenic differences between viral variants included in vaccines are both critical for vaccine development. Prior studies have indicated that HCV genotypes (gts) do not dictate the sensitivity of HCV envelope glycoprotein (E1E2) variants to neutralizing antibodies. However, most of these prior studies under-sampled variants from gts 2–6. Here, we selected a genetically diverse and representative panel of gt 2–6 E1E2 variants, used them to generate HCV pseudoparticles (HCVpp), and measured neutralization of these HCVpp by neutralizing antibodies and HCV-immune plasma from persons infected with gt 1–6 viruses. We found that neutralization results obtained with this gt 2–6 panel were remarkably similar to results obtained with a previously described, antigenically diverse, gt 1-predominant reference panel of 15 HCVpp. These data confirm that, even considering genetically diverse HCV variants across gt 1–6, E1E2 antigenicity is not dictated by gt, and that the previously published panel of 15 HCVpp represents neutralization of all HCV gts with reasonable accuracy.

## Data Availability

All data are available upon request. No new sequences were generated for this study.

## Introduction

The global burden of hepatitis C virus (HCV) is staggering, with an estimated 1 million new infections annually [1]. Up to 50% of those with chronic HCV in the USA are unaware of their infection status, highlighting a critical gap in detection and treatment [2]. Left untreated, HCV can cause hepatocellular carcinoma, cirrhosis, end-stage liver disease and death. Although curative treatment exists, it is not widely accessible [3,4]. Developing a prophylactic vaccine is pivotal in reducing the incidence of new infections and the end-stage complications of HCV [5,6].

The genetic diversity of HCV is a barrier to vaccine development, with eight genotypes (gts) and 67 subtypes [7,8]. One strategy to tackle this diversity is to induce broadly neutralizing antibodies (bNAbs) that target conserved envelope glycoproteins 1 and 2 (E1E2) sites and consequently block infection by genetically diverse HCV isolates [9,16]. A humoral response with early bNAb induction is associated with the natural control of HCV infection in humans, and bNAbs can also prevent HCV infection in animal models [9,13, 17,22].

A clear understanding of the relationship between genetic distance and antigenic differences between HCV variants is critical to facilitate the selection of variants for inclusion in panels measuring the neutralizing breadth of immune sera and variants for inclusion in candidate vaccines. In a study of HCV reinfection after spontaneous viral clearance, repeated infections with viruses carrying bNAb-sensitive E1E2 proteins were associated with induction of bNAbs [23]. Notably, greater genetic distance between re-infecting viruses was not correlated with the induction of broader neutralizing antibody responses. Similarly, prior analyses of panels of genetically diverse HCV variants have shown little association between HCV gt and sensitivity to neutralization [24,26]. These findings indicate that genetic distance between HCV E1E2 variants is not a primary determinant of the antigenic differences between these variants.

We previously selected a neutralization panel of 15 antigenically diverse gt 1a (*n*=8), 1b (*n*=4), 3a (*n*=1), 4a (*n*=1) and 5a (*n*=1) HCV E1E2 variants based on differences in their overall neutralization sensitivity (Tiers 1–4), as well as in patterns of relative sensitivity to seven broadly neutralizing monoclonal antibodies (mAbs) targeting diverse E1E2 epitopes (i.e. antigenic profiles) [26]. As in prior studies, we observed no association between antigenicity and HCV gt. However, a limitation of that study was that most variants analysed were from gt 1. The final set of 15 HCV pseudoparticles (HCVpp) was downselected from an original set of 56 gt 1 variants and only nine gt 2–6 variants. Thus, the number of gt 2–6 variants analysed was small and did not represent the full genetic diversity of those HCV gts, so it remained possible that some antigenic features unique to gt 2–6 variants were not represented by the final panel of 15 E1E2 variants.

To address this limitation, we selected and synthesized 37 genetically diverse and representative gt 2–6 E1E2 variants from global databases. Using the subset of 12 variants that generated highly functional HCVpp, we measured neutralization by the same panels of bNAbs and human plasma samples used to characterize the previously published panel of 15 E1E2 variants. We analysed overall neutralization sensitivity as well as patterns of relative sensitivity to different bNAbs to identify any antigenic features that were unique to the new gt 2–6 E1E2 variants relative to the existing panel.

## Methods

### Cell lines

Single sources of Huh7 human hepatoma [25] and Cluster of Differentiation 81-knockout (CD81-ko) Human Embryo Kidney (HEK) 293T cells [27] (Dr. Joe Grove, University of Glasgow, Glasgow, UK) were used for the production of HCVpp. Cells were grown in Dulbecco’s Modified Essential Medium (Invitrogen, Carlsbad, CA, USA) supplemented with 10% FBS and 0.1 mmol l^−1^ nonessential amino acids (Invitrogen).

### Antibodies

HCV mAbs CBH-7 [11], HC84.26 [10] and HC33.4 [12] were produced by Steven Foung. mAbs AR3A [9] and AR4A [13] were produced by Mansun Law. MAb hAP33 (a chimeric mouse AP33–human crystallizable fragment (Fc) antibody) [16,28] was produced by Arvind Patel, and mAb HCV1 [29] was kindly provided by Yang Wang (MassBiologics, Boston, MA, USA).

### Plasma

Plasma from gt 1–3-infected donors was obtained from the Baltimore Before and After Acute Study of Hepatitis [30]. Plasma samples representing gt 4–6 HCV infections were obtained from the University of Nottingham Trent HCV Cohort study [31]. Plasma was heat inactivated at 56 °C for 30 min prior to use.

### Sequence analysis

Gt 2–6 E1E2 protein sequences were obtained from the National Center for Biotechnology Information (NCBI) GenBank, Los Alamos National Laboratory (LANL) HCV database [32], and the European HCV database (euHCVdb) [33], followed by filtering to remove partial sequences and redundant sequences (exact matches), yielding a total of 824 E1E2 reference sequences. A multiple sequence alignment (MSA) of the sequences was performed using the MAFFT tool (version 7) [34], and candidate sets of panel sequences were aligned to this reference MSA using the MAFFT ‘--add’ option. Sets of candidate gt 2–6 E1E2 panel sequences were generated as before for gt 1 E1E2 panels [26], selecting clade representatives from the reference set to maximize polymorphism coverage for each gt. A phylogenetic tree of the reference sequences was generated using the neighbour-joining method in MAFFT, and the tree was visualized using iTOL [35].

### HCVpp production

Human immunodeficiency virus (HIV) Group-specific antigen (Gag)-packaged HCVpp were generated by Lipofectamine-mediated transfection of HCV E1E2 plasmid, pNL4-3.Luc.R-E- plasmid containing the env-defective HIV proviral genome (NIH AIDS Reagent Program), and pAdVantage (Promega) plasmid into CD81ko-HEK293T cells as described [36]. Mock pseudoparticles (mockpps) generated without E1E2 plasmid were used as a negative control for each transfection.

### HCVpp entry

Huh7 cells (15,000 per well) were plated in 96-well microplates and incubated overnight. Then, 50 µl of HCVpp was added to the Huh7 cells in triplicate, and plates were incubated at 37 °C for 5 h. HCVpp were removed and replaced with phenol-free media, and cells were incubated for 72 h at 37 °C. HCVpp entry was determined by measurement of luciferase activity of cell lysate in relative light units (RLUs).

### Neutralization

Neutralization assays were performed as described previously [36]. mAbs were serially fivefold diluted, starting at a concentration of 100 µg ml^−1^ (leaving the last well as PBS only), and incubated with HCVpp for 1 h at 37 °C before addition to Huh7 target cells in duplicate. HCVpp entry was measured as above. The percentage of neutralization was calculated as [1 − (RLU_mAb_/RLU_PBS_)] × 100, with the PBS RLU values averaged across two plates. Polyclonal human IgG (Thermo Fisher) was used as a negative control. Log_10_ 50% inhibitory concentrations (log_10_IC_50_) were calculated from neutralization curves fit by nonlinear regression [log(inhibitor) versus normalized response, variable slope] in Prism v10 (GraphPad Software). MAb–HCVpp tests that did not reach 50% inhibition were assigned an IC_50_ of 100 µg ml^−1^. Plasma samples were tested at a 1 : 20 dilution. Pooled plasma from ten HCV-negative donors, also at a 1 : 20 dilution, was used as a negative control. Percentage neutralization of each HCVpp was calculated as [1 − (RLU_immune plasma_/RLU_control plasma_)] × 100.

## Results

### Selection of representative gt 2–6 HCV E1E2 variants for functional analysis

**Fig. 1.**
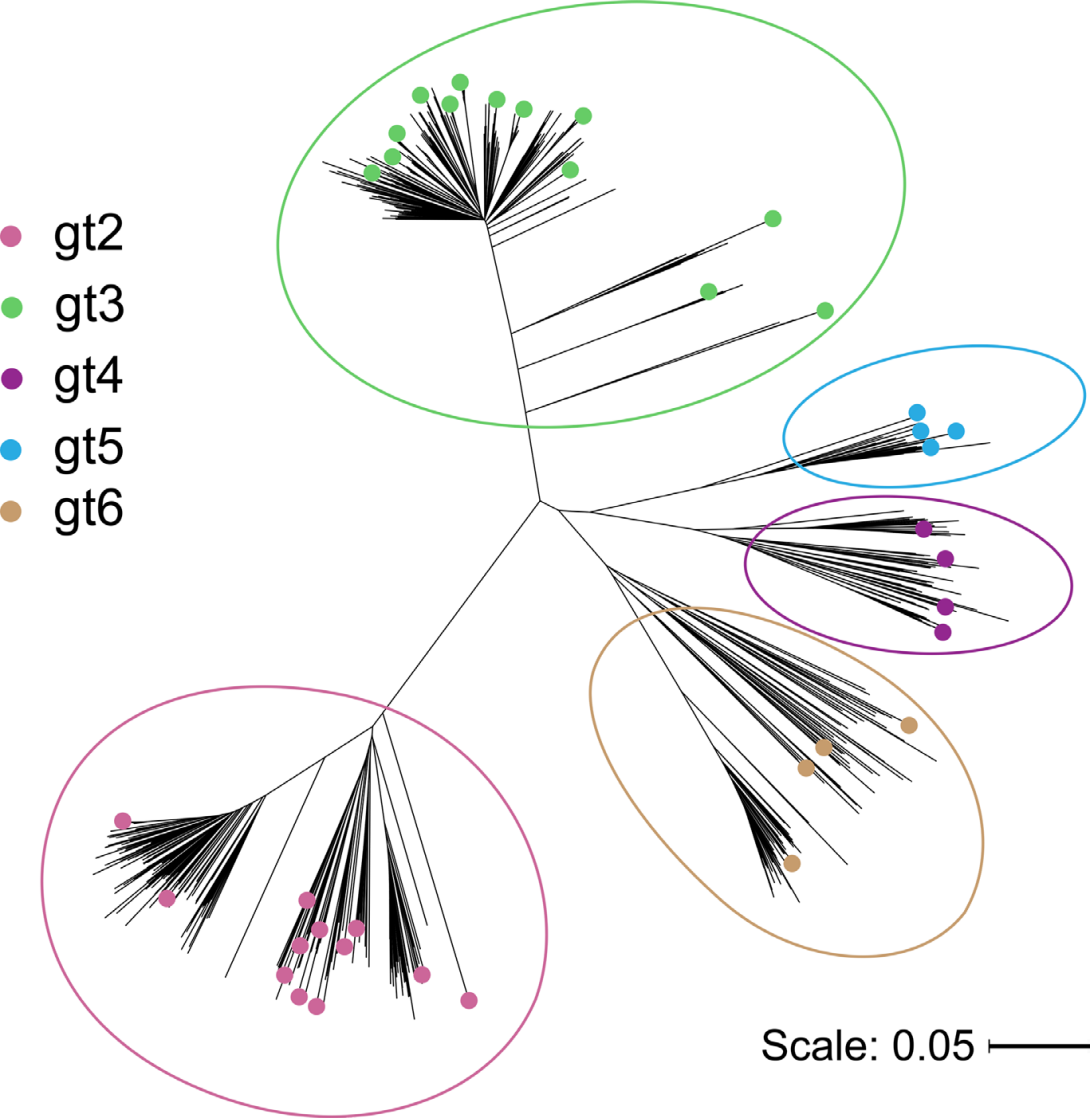
Phylogenetic tree of reference and selected panel E1E2 sequences. A set of gts 2–6 E1E2 reference sequences (*N*=824) was processed with the MAFFT tool to generate an MSA and phylogenetic tree. The tree was visualized using iTOL. Selected panel sequences are indicated by coloured circles, and gts are circled for reference.

We downloaded all available gt 2–6 full E1E2 sequences from the NCBI GenBank, LANL [32] and euHCVdb [33] databases (*n*=824). Using the aligned set of 824 E1E2 amino acid sequences, we used a custom script to identify amino acid polymorphisms at each position in E1E2 that were present in 2–80% of database isolates. This frequency range was chosen to focus on variable sites, while capturing polymorphisms that occur commonly enough to represent genuine circulating diversity (≥2% of isolates), excluding extremely rare substitutions that likely reflect isolated mutations or PCR errors. Based on this filtered dataset, we then identified 37 representative E1E2 variants (Table S1, available in the online Supplementary Material) expressing 97% of all common gt 2–6 polymorphisms and representative of diverse gt 2–6 phylogenetic clades (Fig. 1). We synthesized codon-optimized nucleotide sequences representing each of these amino acid sequences and cloned these into a mammalian expression vector, along with an N-terminal tissue plasminogen activator signal sequence.

### Wide variation in hepatoma cell entry of HCVpp gt 2–6 HCVpp

**Fig. 2.**
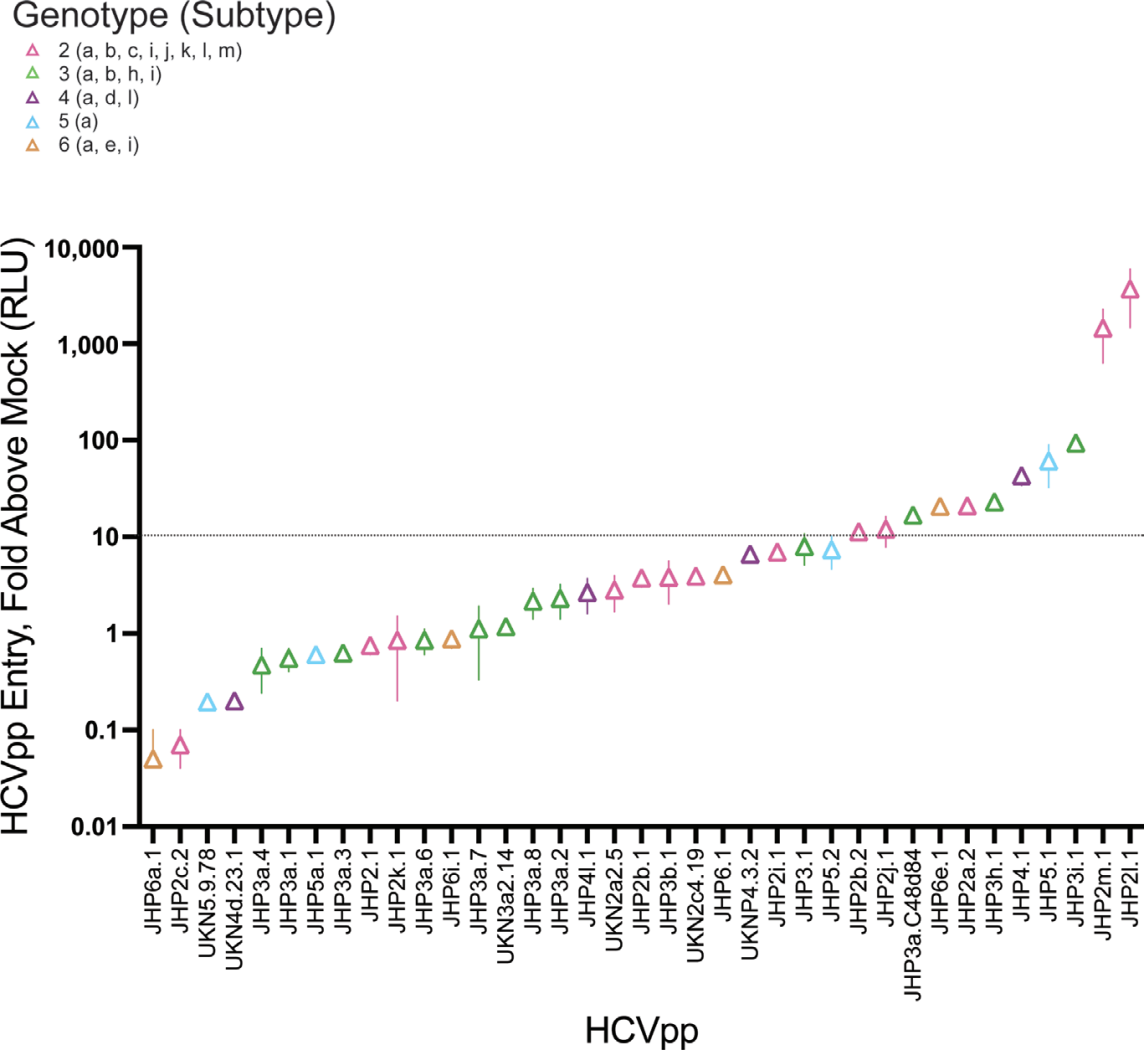
Wide variation in hepatoma cell entry of gt 2–6 HCVpp. Hepatoma cell entry of 37 HCVpp is expressed as the fold increase in RLUs compared with the background entry of mockpp. Data represent 2–6 independent experiments for each HCVpp, performed in triplicate. Symbols indicate means, and whiskers indicate the sem. A threshold of ten-fold above mockpp entry is indicated with a dotted line.

Using a standard approach, we generated HCVpp by transfecting each E1E2 expression plasmid along with a pNL4-3.Luc.R-E- expression plasmid containing the *env*-defective HIV proviral genome and collecting HCVpp-containing supernatants [36]. We measured the entry of HCVpp into Huh7 hepatoma cells to assess the relative function of each E1E2 glycoprotein (Fig. 2). Pseudoparticles lacking E1E2, known as mockpps, were tested in parallel to quantify nonspecific entry. Specific entries were calculated as a ratio of HCVpp entry relative to mockpp entry, with a predetermined threshold of ten-fold above mock defined as optimally functional for neutralization testing. Out of the 37 HCVpp, 11 (29.73%) exhibited specific entry exceeding the ten-fold cut-off in at least two production runs. One HCVpp (JHP5.2) was ten-fold above mock in only one production run but was carried forward to neutralization experiments. We selected these 12 HCVpp for subsequent neutralization testing; they included gts 2 (*n*=5), 3 (*n*=3), 4 (*n*=1), 5 (*n*=2) and 6 (*n*=1). The specific entry of HCVpp did not segregate by gt. Although some genetic diversity was lost by downselection from the original set of 37 E1E2 variants, this set of 12 highly functional HCVpp expressed 79% of all commonly observed gt 2–6 E1E2 polymorphisms.

### Distribution of neutralization sensitivity of gt 2–6 HCVpp parallels the published panel of 15 HCVpp

**Fig. 3.**
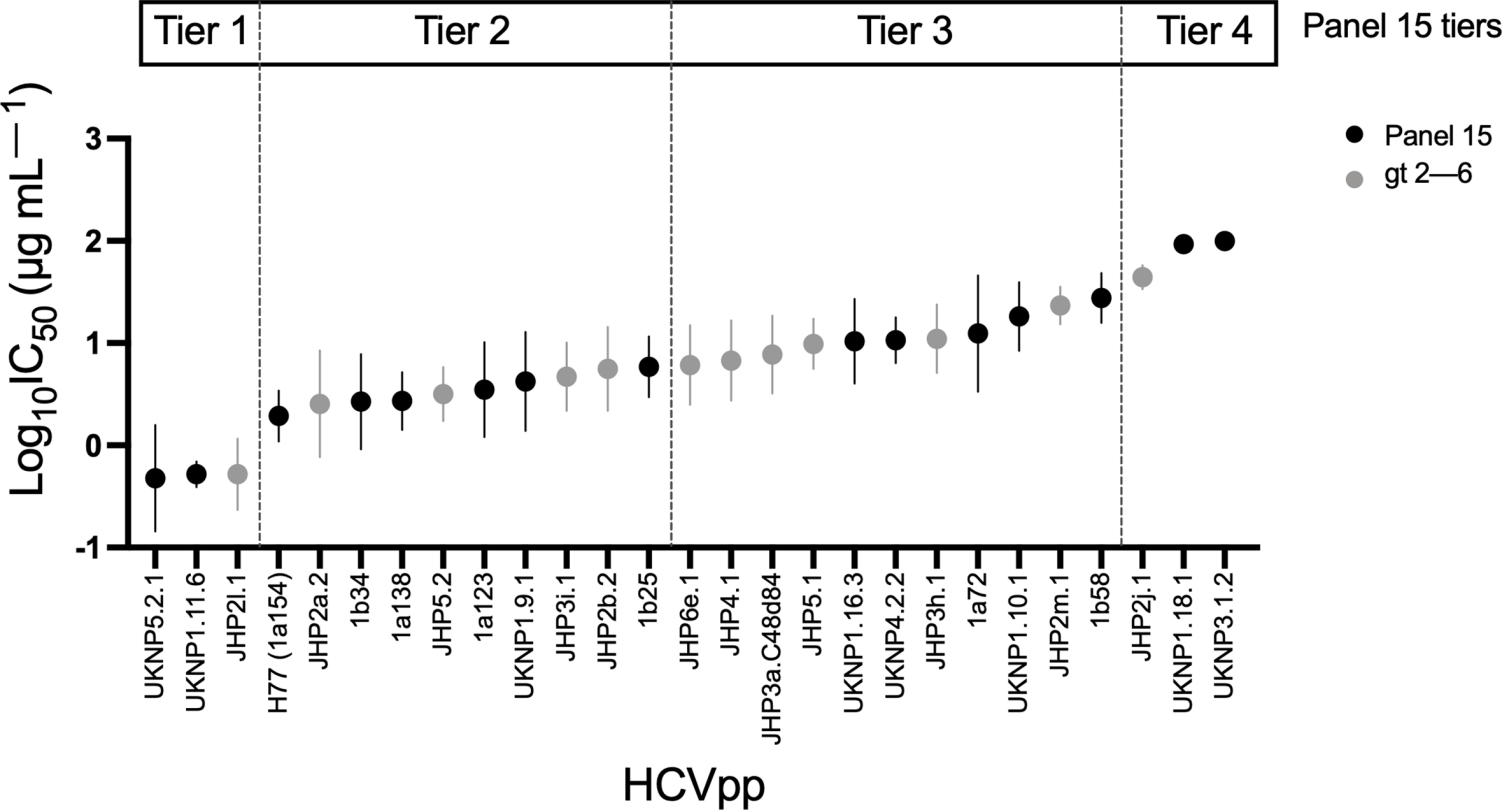
Distribution of neutralization sensitivity of gt 2–6 HCVpp parallels the published panel of 15 HCVpp. Neutralization of gt 2–6 HCVpp was measured with seven bNAbs targeting diverse epitopes across E1E2. For reference, previously published neutralization data for the standard panel of 15 HCVpp from Salas *et al*. [26] is included (‘panel 15’, black circles). Data represent two independent experiments for each mAb-HCVpp combination, performed in duplicate. The symbols indicate means, and the whiskers indicate the sem. HCVpp are arranged from most to least neutralization sensitive based on mean log_10_IC_50_ and divided into four tiers based on sd from the mean of the standard panel of 15 HCVpp.

We measured the neutralization of this panel of 12 gt 2–6 HCVpp. The neutralization of each HCVpp was tested using serial dilutions of the same seven bNAbs used to characterize the published 15 HCVpp panel, chosen for their ability to bind to a range of neutralizing epitopes across the E2 glycoprotein or E1E2 heterodimer (Table S2) [9,13,16, 29]. These seven mAbs recognize five distinct antigenic sites, including Domain B/AR3 (mAb AR3A), Domain C (mAb CBH-7), Domain D (mAb HC84.26), Domain AR4 (mAb AR4A) and Domain E/AS412 (HC33.4, HCV1 and hAP33). All mAbs were isolated from humans infected with HCV, except HCV1, which was generated by immunizing a transgenic mouse expressing human antibody genes [29], and hAP33, which was generated by immunization of a WT mouse [16], and then subsequently produced as a mouse–human chimera (i.e. its variable heavy and variable light chains grafted onto a human IgG1 Fc backbone) [28]. Neutralization of the 12 gt 2–6 HCVpp was compared with the previously published neutralization results obtained with the panel of 15 HCVpp (Fig. 3). To confirm reproducibility, neutralization of four control HCVpp representing Tiers 1–4 of the published panel of 15 HCVpp [UKNP1.11.6, H77 (1a154), 1a72 and UKNP1.18.1] was re-tested in parallel with the 12 gt 2–6 HCVpp. As expected, neutralization of the four control HCVpp was consistent with previously published data for the same HCVpp (Fig. S1).

We observed a wide range of neutralization sensitivity across the 12 gt 2–6 HCVpp. We ranked the HCVpp from lowest to highest mean log_10_IC_50_ across the seven bNAbs. The mAbs HCV1 and hAP33 bind very similar epitopes on E2, so we averaged the log_10_IC_50_ values for these two mAbs, giving HCV1 and hAP33 each half of the weight of the other five bNAbs in our analysis. The HCVpp were separated into four tiers based on the normal distribution of their mean log_10_IC_50_ values. The distribution of neutralization sensitivity of the 12 gt 2–6 HCVpp was similar to the distribution of the previously published panel of 15 HCVpp, with one HCVpp (8.33%) in Tier 1, four HCVpp (33.33%) in Tier 2, five HCVpp (41.67%) in Tier 3 and two HCVpp (16.67%) in Tier 4. When IC_50_ values for each antibody–HCVpp pair were averaged (with HCV1 and AP33 combined as a single entry), the mean IC_50_ was 27.42 µg ml^−1^. The average IC_50_ across all mAbs for each HCVpp ranged from 1.64 to 61.70 µg ml^−1^ (Table S2). The panel of 15 HCVpp had very similar IC_50_ values with the same mAbs (mean=35.94 µg ml^−1^; range across HCVpp: 0.63 to >100 µg ml^−1^) [26]. For each mAb, we analysed the relationship between the number of substitutions in mAb epitopes for each E1E2 variant and neutralization resistance of the HCVpp (Fig. S2). For HCV1 and AP33, we observed a trend towards greater resistance with a larger number of substitutions, although these differences were not statistically significant. For the remaining mAbs, there was no apparent relationship between the number of epitope substitutions and mAb resistance, which is consistent with prior observations that polymorphisms distant from epitopes play a dominant role in neutralization resistance [37,39]. Together, these data demonstrate that the gt 2–6 HCVpp have a wide range of neutralization sensitivity, mirroring the previously published panel of 15 HCVpp.

### Neutralization breadth of bNAbs is very similar when measured with either the panel of 12 gt 2–6 HCVpp or the published panel of 15 HCVpp

The neutralizing breadth (i.e. per cent of HCVpp neutralized >50%) measured for each bNAb at IC_50_ thresholds of 100, 10 or 1 µg ml^−1^ was somewhat higher for HC84.26 and AR3A mAbs when quantitated using the gt 2–6 HCVpp panel rather than the panel of 15 HCVpp, but notably, average neutralizing breadth across all mAbs was remarkably similar when measured with either HCVpp panel (Table 1). For example, at an IC_50_ threshold of 10 µg ml^−1^, an average of 63% of the variants in the gt 2–6 panel and 60% of the variants in the panel of 15 HCVpp were neutralized.

**Table 1.** Neutralizing breadth of reference monoclonal antibodies measured using gt 2–6 HCVpp or the previously published panel of 15 HCVpp (from Salas *et al*. [26])

mAbs	HCVpp panel	HCVpp no.	Neutralizing breadth, %*		
			100 µg ml^−1^	10 µg ml^−1^	1 µg ml^−1^
hAP33	gt 2–6	12	92	75	25
	Panel of 15	15	93	80	47
HCV1	gt 2–6	12	92	75	17
	Panel of 15	15	87	87	40
AR4A	gt 2–6	12	92	83	42
	Panel of 15	15	87	80	33
HC84.26	gt 2–6	12	100	92	67
	Panel of 15	15	73	67	33
AR3A	gt 2–6	12	100	75	33
	Panel of 15	15	60	53	13
HC33.4	gt 2–6	12	58	33	8
	Panel of 15	15	73	40	27
CBH-7	gt 2–6	12	25	8	0
	Panel of 15	15	33	13	13
Average	gt 2–6	12	80	63	27
Average	Panel of 15	15	72	60	29

*Per cent of all HCVpp in each panel neutralized with an IC_50_ less than the indicated value.

### Neutralization breadth of gt 2–6-infected plasma is similar when measured with either the panel of 12 gt 2–6 HCVpp or the published panel of 15 HCVpp

**Fig. 4.**
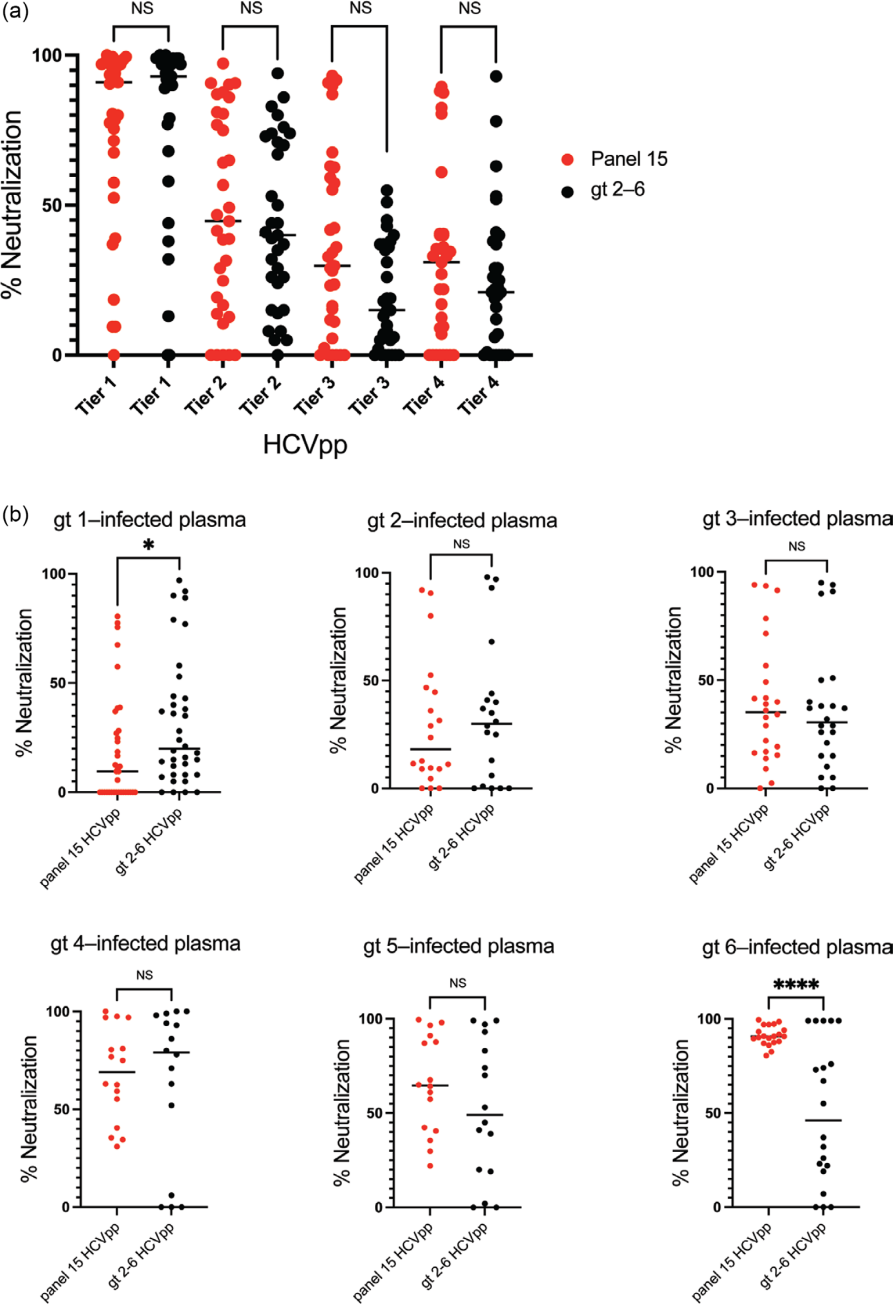
Overall sensitivity to HCV-immune plasma of Tier 1–4 variants from gt 2–6 HCVpp and the published panel of 15 HCVpp are similar. (**a**) Per cent neutralization of each tier of the gt 2–6 HCVpp by plasma at 1 : 20 dilution from 33 individuals infected with gt 1–6 HCV, measured in duplicate, compared to published neutralization values for the panel of 15 HCVpp measured with the same plasma samples (Salas *et al*. [26]). Each point indicates the mean neutralization of all HCVpp in the indicated tier by a single plasma sample. Neutralization values <0 were normalized to 0. Horizontal lines indicate medians. Groups were compared by the Kruskal–Wallis test, with adjustment for multiple comparisons. (**b**) Neutralization of each HCVpp panel by HCV-immune plasma, with results segregated by the infecting gt of each participant. Each point indicates the mean neutralization of all HCVpp in each tier by a single plasma sample. Horizontal lines indicate medians. Non-normally distributed groups (gt 1–4 and gt 6) were compared by Kolmogorov–Smirnov test. The normally distributed group (gt 5) was compared by an unpaired t-test. ns, not significant; ^∗^*P*<0.05, ^∗∗∗∗^*P*<0.0001.

Using 33 plasma samples from persons infected with HCV, we tested neutralizing breadth using the gt 2–6 HCVpp panel (Table S3). We then compared these results with previously published data, which were acquired with the panel of 15 HCVpp and the same plasma samples (Fig. 4). The plasma samples were isolated from persons infected with gt 1 (*n*=9), 2 (*n*=5), 3 (*n*=6), 4 (*n*=4), 5 (*n*=4) or 6 (*n*=5) infections. Using plasma from all six gts, there were no significant differences between neutralization of Tier 1, 2, 3 or 4 HCVpp in the panel of gt 2–6 HCVpp relative to HCVpp in the same tiers from the panel of 15 HCVpp (Figs 4a and S3). As expected, when we compared the neutralization of Tier 1–4 HCVpp by gt 2–6-infected plasma, we observed significant differences between each tier, with the exception of Tiers 3 and 4, which showed similar neutralization sensitivity (Fig. S4).

We then compared neutralization of the panel of gt 2–6 HCVpp and the panel of 15 HCVpp with plasma segregated by the HCV gt infecting each participant. There were no differences between panels in neutralization by gt 2-, 3-, 4- or 5-infected plasma. Notably, however, relative to the panel of 15 HCVpp, the panel of gt 2–6 HCVpp was slightly more sensitive to neutralization by gt 1-infected plasma and significantly more resistant to neutralization by gt 6-infected plasma (Figs 4b and S5). Overall, very similar neutralization results were obtained when testing HCV-immune plasma with either the panel of gt 2–6 HCVpp or the published panel of 15 HCVpp.

## Discussion

Producing a vaccine for HCV demands an accurate assessment of the neutralizing breadth of antibodies elicited by the vaccine, and an understanding of the antigenic differences between potential vaccine variants. We previously identified a standardized panel of 15 antigenically diverse gt 1a, 1b, 3a, 4a and 5a HCV E1E2 variants for use in neutralization assays [26], but some concern remained that this panel did not fully represent the antigenic diversity of all gts. Therefore, we focused in this study on identifying genetically diverse and representative gt 2–6 E1E2 variants, and we compared the antigenic characteristics of these variants with those described for the published panel of 15 HCVpp. We observed remarkable similarity between the new gt 2–6 HCVpp panel and the existing 15 HCVpp panel in the range and magnitude of neutralization sensitivity to bNAbs or HCV-immune plasma. Neutralization breadth for each of the reference bNAbs was very similar when measured with either the published panel of 15 HCVpp or the new gt 2–6 panel. The only notable difference between the panels was in sensitivity to neutralization by plasma from gt 6-infected persons, with the new gt 2–6 panel displaying greater resistance to neutralization relative to the published panel of 15 HCVpp. The underlying mechanism is unclear, but we would speculate that this may indicate that the NAbs induced by gt 6 infection may target some unique epitopes relative to the NAbs induced by gt 1–5 infections. Further work to understand this phenomenon is warranted, and inclusion of these gt 2–6 variants should be considered for studies of neutralization in gt 6-infected individuals.

Neutralization results with the two panels are similar, even though the original panel of 15 HCVpp is dominated by gt 1 variants, while the new panel is focused exclusively on gt 2–6 variants. The original panel of 15 E1E2 variants expresses only 60% of common gt 2–6 polymorphisms, while the gt 2–6 panel expresses 79% of those polymorphisms. The similarity in neutralization results between panels supports prior studies [17,24, 38, 40, 41] indicating that the majority of E1E2 amino acid differences across gts are irrelevant to heterologous NAb sensitivity. Given the antigenic similarity between these panels, and the extensive data already generated across multiple laboratories using the original panel of 15 HCVpps, we would propose that there is no need to add these new gt 2–6 variants to the existing 15 HCVpp panel for standardized neutralization testing. These data provide further confirmation that neutralization data obtained with the panel of 15 HCVpp are generally representative of neutralization of all HCV variants from gts 1–6.

There are some limitations to this study. First, relatively few gt 2–6 E1E2 sequences are available in global databases. Even though all available sequences were analysed for this study, there may yet be variants in nature from gts 2–6, or other gts, with unique antigenic characteristics. Work should continue to sequence new variants from these still under-sampled gts. While we were careful to use bNAbs that target a variety of neutralizing epitopes, and we also tested neutralization with polyclonal sera that contain a variety of biologically relevant NAbs, it is possible that bNAbs isolated in the future might target new epitopes, which could result in different results between the two HCVpp panels.

A vaccine for HCV is urgently needed, and development of this vaccine will require accurate measurement of antibody neutralization breadth and understanding of the antigenic differences between E1E2 variants. We have characterized a genetically diverse and representative set of gt 2–6 E1E2 variants and found that neutralization results obtained with these variants very closely mirror results obtained with our previously published gt 1-dominant panel of 15 HCVpp. These data confirm that neutralization results obtained with the standardized panel of 15 HCVpp are generally representative of neutralization of all HCV gts.

## Supplementary material

10.1099/jgv.0.002201 Uncited Supplementary Material 1.

## Abbreviations

bNAbs: broadly neutralizing antibodies
euHCVdb: european HCV database
Fc: crystallizable fragment
gt: genotype
HCV: hepatitis C virus
HCVpps: hepatitis C virus pseudoparticles
HIV: human immunodeficiency virus
LANL: Los Alamos National Laboratory
log_10_IC_50_: log_10_ 50% inhibitory concentrations
mAbs: monoclonal antibodies
mockpp: mock pseudoparticle
MSA: multiple sequence alignment
NCBI: National Center for Biotechnology Information
RLU: relative light unit

## References

[1] World Health Organization (2024). Global Hepatitis Report.

[2] Terrault NA (2019). Hepatitis C elimination: challenges with under-diagnosis and under-treatment. F1000Res.

[3] Kanwal F, Kramer J, Asch SM, Chayanupatkul M, Cao Y (2017). Risk of *Hepatocellular* cancer in HCV patients treated with direct-acting antiviral agents. Gastroenterology.

[4] Hamdane N, Jühling F, Crouchet E, El Saghire H, Thumann C (2019). HCV-induced epigenetic changes associated with liver cancer risk persist after sustained virologic response. Gastroenterology.

[5] Bailey JR, Barnes E, Cox AL (2019). Approaches, progress, and challenges to hepatitis C vaccine development. Gastroenterology.

[6] Bartenschlager R, Baumert TF, Bukh J, Houghton M, Lemon SM (2018). Critical challenges and emerging opportunities in hepatitis C virus research in an era of potent antiviral therapy: considerations for scientists and funding agencies. Virus Res.

[7] Simmonds P, Becher P, Bukh J, Gould EA, Meyers G (2017). ICTV virus taxonomy profile: flaviviridae. J Gen Virol.

[8] Smith DB, Bukh J, Kuiken C, Muerhoff AS, Rice CM (2014). Expanded classification of hepatitis C virus into 7 genotypes and 67 subtypes: updated criteria and genotype assignment web resource. Hepatology.

[9] Law M, Maruyama T, Lewis J, Giang E, Tarr AW (2008). Broadly neutralizing antibodies protect against hepatitis C virus quasispecies challenge. Nat Med.

[10] Keck Z, Xia J, Wang Y, Wang W, Krey T (2012). Human monoclonal antibodies to a novel cluster of conformational epitopes on HCV E2 with resistance to neutralization escape in a genotype 2a isolate. PLoS Pathog.

[11] Hadlock KG, Lanford RE, Perkins S, Rowe J, Yang Q (2000). Human monoclonal antibodies that inhibit binding of hepatitis C virus E2 Protein to CD81 and recognize conserved conformational epitopes. J Virol.

[12] Keck Z, Wang W, Wang Y, Lau P, Carlsen THR (2013). Cooperativity in virus neutralization by human monoclonal antibodies to two adjacent regions located at the amino terminus of hepatitis C virus E2 glycoprotein. J Virol.

[13] Giang E, Dorner M, Prentoe JC, Dreux M, Evans MJ (2012). Human broadly neutralizing antibodies to the envelope glycoprotein complex of hepatitis C virus. Proc Natl Acad Sci USA.

[14] Johansson DX, Voisset C, Tarr AW, Aung M, Ball JK (2007). Human combinatorial libraries yield rare antibodies that broadly neutralize hepatitis C virus. Proc Natl Acad Sci USA.

[15] Bailey JR, Flyak AI, Cohen VJ, Li H, Wasilewski LN (2017). Broadly neutralizing antibodies with few somatic mutations and hepatitis C virus clearance. JCI Insight.

[16] Owsianka A, Tarr AW, Juttla VS, Lavillette D, Bartosch B (2005). Monoclonal antibody AP33 defines a broadly neutralizing epitope on the hepatitis C virus E2 envelope glycoprotein. J Virol.

[17] Osburn WO, Snider AE, Wells BL, Latanich R, Bailey JR (2014). Clearance of hepatitis C infection is associated with the early appearance of broad neutralizing antibody responses. Hepatology.

[18] Pestka JM, Zeisel MB, Bläser E, Schürmann P, Bartosch B (2007). Rapid induction of virus-neutralizing antibodies and viral clearance in a single-source outbreak of hepatitis C. Proc Natl Acad Sci USA.

[19] Meuleman P, Bukh J, Verhoye L, Farhoudi A, Vanwolleghem T (2011). In vivo evaluation of the cross-genotype neutralizing activity of polyclonal antibodies against hepatitis C virus. Hepatology.

[20] Keck Z, Wang Y, Lau P, Lund G, Rangarajan S (2016). Affinity maturation of a broadly neutralizing human monoclonal antibody that prevents acute hepatitis C virus infection in mice. Hepatology.

[21] Morin TJ, Broering TJ, Leav BA, Blair BM, Rowley KJ (2012). Human monoclonal antibody HCV1 effectively prevents and treats HCV infection in chimpanzees. PLoS Pathog.

[22] Kinchen VJ, Zahid MN, Flyak AI, Soliman MG, Learn GH (2018). Broadly neutralizing antibody mediated clearance of human hepatitis C virus infection. Cell Host Microbe.

[23] Frumento N, Figueroa A, Wang T, Zahid MN, Wang S (2022). Repeated exposure to heterologous hepatitis C viruses associates with enhanced neutralizing antibody breadth and potency. J Clin Invest.

[24] Carlsen THR, Pedersen J, Prentoe JC, Giang E, Keck Z-Y (2014). Breadth of neutralization and synergy of clinically relevant human monoclonal antibodies against HCV genotypes 1a, 1b, 2a, 2b, 2c, and 3a. Hepatology.

[25] Urbanowicz RA, McClure CP, Brown RJP, Tsoleridis T, Persson MAA (2016). A diverse panel of hepatitis C virus glycoproteins for use in vaccine research reveals extremes of monoclonal antibody neutralization resistance. J Virol.

[26] Salas JH, Urbanowicz RA, Guest JD, Frumento N, Figueroa A (2022). An antigenically diverse, representative panel of envelope glycoproteins for hepatitis C virus vaccine development. Gastroenterology.

[27] Kalemera MD, Capella-Pujol J, Chumbe A, Underwood A, Bull RA (2021). Optimized cell systems for the investigation of hepatitis C virus E1E2 glycoproteins. J Gen Virol.

[28] Pantua H, Diao J, Ultsch M, Hazen M, Mathieu M (2013). Glycan shifting on hepatitis C virus (HCV) E2 glycoprotein is a mechanism for escape from broadly neutralizing antibodies. J Mol Biol.

[29] Broering TJ, Garrity KA, Boatright NK, Sloan SE, Sandor F (2009). Identification and characterization of broadly neutralizing human monoclonal antibodies directed against the E2 envelope glycoprotein of hepatitis C virus. J Virol.

[30] Cox AL, Netski DM, Mosbruger T, Sherman SG, Strathdee S (2005). Prospective evaluation of community‐acquired acute‐phase hepatitis C virus infection. Clin Infect Dis.

[31] Mohsen AH, Trent HCVSG (2001). The epidemiology of hepatitis C in a UK health regional population of 5.12 million. Gut.

[32] Kuiken C, Yusim K, Boykin L, Richardson R (2005). The Los Alamos hepatitis C sequence database. Bioinformatics.

[33] Combet C, Garnier N, Charavay C, Grando D, Crisan D (2007). euHCVdb: the European hepatitis C virus database. Nucleic Acids Res.

[34] Katoh K, Standley DM (2013). MAFFT multiple sequence alignment software version 7: improvements in performance and usability. Mol Biol Evol.

[35] Letunic I, Bork P (2021). Interactive Tree Of Life (iTOL) v5: an online tool for phylogenetic tree display and annotation. Nucleic Acids Res.

[36] Bailey JR, Urbanowicz RA, Ball JK, Law M, Foung SKH (2019). Standardized method for the study of antibody neutralization of HCV pseudoparticles (HCVpp). Methods Mol Biol.

[37] Bailey JR, Wasilewski LN, Snider AE, El-Diwany R, Osburn WO (2015). Naturally selected hepatitis C virus polymorphisms confer broad neutralizing antibody resistance. J Clin Invest.

[38] El-Diwany R, Cohen VJ, Mankowski MC, Wasilewski LN, Brady JK (2017). Extra-epitopic hepatitis C virus polymorphisms confer resistance to broadly neutralizing antibodies by modulating binding to scavenger receptor B1. PLoS Pathog.

[39] Augestad EH, Castelli M, Clementi N, Ströh LJ, Krey T (2020). Global and local envelope protein dynamics of hepatitis C virus determine broad antibody sensitivity. Sci Adv.

[40] Bankwitz D, Bahai A, Labuhn M, Doepke M, Ginkel C (2021). Hepatitis C reference viruses highlight potent antibody responses and diverse viral functional interactions with neutralising antibodies. Gut.

[41] Urbanowicz RA, McClure CP, Brown RJP, Tsoleridis T, Persson MAA (2016). A diverse panel of hepatitis C virus glycoproteins for use in vaccine research reveals extremes of monoclonal antibody neutralization resistance. J Virol.

