# Supplementary material for: Antigenicity of key hepatitis C virus E1E2 glycoprotein neutralizing sites is genotype independent
Source: J Gen Virol. 2026 Jan 13;107(1):002201. doi: 10.1099/jgv.0.002201 (PMC12798819; doi:10.1099/jgv.0.002201)
Supplement: Uncited Supplementary Material 1. [file jgv-107-02201-s001.pdf]

Supplementary Materials

Supplementary Table 1. E1E2 GenBank accession numbers, and genotypes/subtypes.

E1E2 Tested for Entry

HCVpp	Accession #	Subtype
JHP2.1	AGV23523.1	2
UKN2a2.5	AAW65872.1	2a
JHP2a.2	AHK22794.1	2a
JHP2b.1	AFQ54458.1	2b
JHP2b.2	BAK61633.1	2b
JHP2c.2	JX227949	2c
UKN2c4.19	ANO46563.1	2c
JHP2i.1	AGV23515.1	2i
JHP2j.1	AFD18566.1	2j
JHP2k.1	AEI00314.1	2k
JHP2l.1	AGV23519.1	2l
JHP2m.1	AFD18564.1	2m
JHP3.1	AOF41131.1	3
JHP3a.1	AFQ53027.1	3a
UKN3a2.14	AAY41630.1	3a
JHP3a.2	AAY41660.1	3a
JHP3a.3	ABK41457.1	3a
JHP3a.4	AFQ54318.1	3a
JHP3a.C48d84	OK502909.1.	3a
JHP3a.6	AOF41176.1	3a

JHP3a.7	AFI23785.1	3a
JHP3a.8	AOF41199.1	3a
JHP3b.1	D49374	3b
JHP3h.1	AFD18579.1	3h
JHP3i.1	AFN53804.1	3i
JHP4.1	AMR44618.1	4
UKN4d.23.1	ABK41464.1	4d
UKNP4.3.2	UKNP4.3.2	4
JHP4l.1	AFN53794.1	4l
JHP5.1	AMB38795.1	5
JHP5a.1	AAS92942.1	5a
JHP5.2	AMR44608.1	5
UKN5.9.78	ABK41470.1	5
JHP6.1	DQ278892	6
JHP6a.1	AAS92947.1	6a
JHP6e.1	AJF19143.1	6e
JHP6i.1	ABI36965.1	6i

E1E2 Tested for Neutralization

HCVpp	Accession #	Subtype
JHP2a.2	AHK22794.1	2a
JHP2b.2	BAK61633.1	2b

JHP2j.1	AFD18566.1	2j
JHP2l.1	AGV23519.1	2l
JHP2m.1	AFD18564.1	2m
JHP3a.C48d84	OK502909.1.	3a
JHP3h.1	AFD18579.1	3h
JHP3i.1	AFN53804.1	3i
JHP4.1	AMR44618.1	4
JHP5.1	AMB38795.1	5
JHP5.2	AMR44608.1	5
JHP6e.1	AJF19143.1	6e

Supplementary Table 2. 50% inhibitory concentrations (IC_{50}s) of 7 mAbs targeting diverse epitopes across the panel of gt 2-6 E1E2. HCV1 and AP33 bind highly similar epitopes so IC_{50}s of these two mAbs were averaged for each HCVpp.

	IC_{50} (ug/mL)											
	tier 1	tier 2				tier 3					tier 4	
mAb	JHP2l.1	JHP2a.2	JHP5.2	JHP3i.1	JHP2b.2	JHP4.1	JHP6e.1	JHP3a.C48d84	JHP5.1	JHP3h.1	JHP2m.1	JHP2j.1
HCV1	1.01	0.17	4.57	1.68	100.00	5.55	0.40	1.45	11.30	2.29	4.32	31.26
HC84.26	0.09	0.07	0.35	2.45	0.52	0.61	0.15	0.11	0.69	1.08	9.10	20.69
AR3A	0.02	0.50	0.18	2.26	0.72	5.10	1.65	10.25	4.16	8.08	13.14	14.28
HC33.4	3.20	100.00	0.11	16.49	7.81	100.00	49.56	100.00	6.99	100.00	41.31	100.00
CBH-7	4.22	100.00	30.98	100.00	15.32	100.00	100.00	100.00	100.00	100.00	100.00	100.00
AP33	0.70	0.08	0.11	1.10	100.00	4.01	1.79	5.07	11.20	3.51	9.17	39.23
AR4A	1.42	0.18	0.47	0.68	0.72	1.31	1.64	0.69	4.22	4.45	16.13	100.00
HCV1/AP33	0.86	0.13	2.34	1.39	100.00	4.78	1.10	3.26	11.25	2.90	6.75	35.25

Supplementary Table 3. Percent neutralization of HCVpp by a 1:20 dilution of 33 plasma samples from genotype 1-6-infected participants, with participant ID numbers matching those used in Salas, et al. (1)

Infecting Genotype	subject	Infection Outcome	HCVpp Tier															
			1			2				3						4		
			JHP2l.1	1.11.6 (repeat)	1a154 (repeat)	JHP2a.2	JHP5.2	JHP3i.1	JHP2b.2	JHP4.1	JHP6e.1	JHP3a.C48d84	JHP5.1	1a72 (repeat)	JHP3h.1	JHP2m.1	1.18.1 (repeat)	JHP2j.1
gt 1	1	Clearance	89	85	71	43	27	58	45	45	19	20	34	65	-6	29	29	19
	2	Clearance	88	85	64	48	42	47	58	39	15	26	32	33	-12	17	6	-57
	3	Clearance	78	58	52	27	25	48	16	35	16	7	-2	33	-38	22	26	4
	4	Clearance	86	76	61	-6	10	-7	53	9	23	-19	2	22	-9	-5	12	-7
	5	Persistence	65	92	45	4	13	6	22	17	21	-66	-31	38	-17	-10	-38	-40
	6	Persistence	74	44	38	4	20	5	6	13	20	31	-13	7	23	1	-21	-45
	7	Persistence	47	41	28	3	20	0	-1	3	10	-10	21	13	-3	9	-7	-85
	8	Persistence	-80	-12	16	11	7	-9	7	16	44	13	13	22	-6	29	26	-1
	9	Persistence	16	30	20	-15	-22	-7	-27	34	-2	-32	7	3	9	5	14	-9
gt 2	10	Persistence	96	81	68	71	45	50	55	64	56	14	26	39	47	16	10	25
	11	Persistence	94	79	65	60	19	26	74	55	44	33	23	30	23	31	22	44
	12	Persistence	86	67	48	51	22	29	50	34	29	5	23	26	45	21	12	44
	13	Persistence	57	35	11	15	46	5	35	4	17	2	16	19	25	10	5	-3
	14	Persistence	-20	9	13	14	2	-9	0	5	9	-33	-5	5	10	2	20	17
gt 3	15	Persistence	91	87	77	67	40	34	19	54	34	34	30	62	36	43	45	56
	16	Persistence	92	83	63	62	41	41	37	50	49	40	28	40	22	30	40	9
	17	Persistence	90	77	54	46	32	36	23	40	36	51	19	37	29	27	16	44
	18	Persistence	88	79	63	19	3	7	25	27	13	12	2	21	17	2	-12	29
	19	Persistence	38	38	40	2	-7	5	9	3	26	-11	-27	3	-36	-17	-24	-4
	20	Persistence	17	41	39	-18	25	9	15	0	-5	-5	22	20	37	4	-24	29
gt 4	21	Persistence	100	95	94	51	86	95	90	74	89	85	94	88	-364	90	77	95
	23	Persistence	98	90	87	59	74	64	86	84	72	75	62	74	-366	60	53	45
	24	Persistence	99	93	82	75	88	84	96	96	61	36	70	69	-232	81	83	75
	25	Persistence	100	99	100	94	95	99	96	96	61	36	70	69	-232	81	83	75
gt 5	26	Persistence	97	34	29	75	73	76	73	78	79	-10	50	-2	30	0	-26	40
	27	Persistence	93	40	9	49	28	82	-4	88	-31	-30	27	-55	-43	-28	36	19
	28	Persistence	99	41	65	63	84	71	60	79	-43	-22	42	18	41	28	31	79
	29	Persistence	99	93	86	77	85	79	91	46	78	0	57	37	-251	63	14	19
gt 6	31	Persistence	99	96	83	63	85	82	74	87	54	-23	8	29	5	-14	3	29
	32	Persistence	99	93	82	75	88	84	36	-58	51	-12	45	-28	-311	-17	18	-74
	33	Persistence	99	93	62	56	81	90	68	63	69	-8	34	22	-344	10	-43	29
	34	Persistence	99	52	52	80	33	77	77	77	65	36	42	29	57	22	-22	22
	35	Persistence	99	28	62	55	76	88	72	93	28	-3	61	52	4	32	20	15

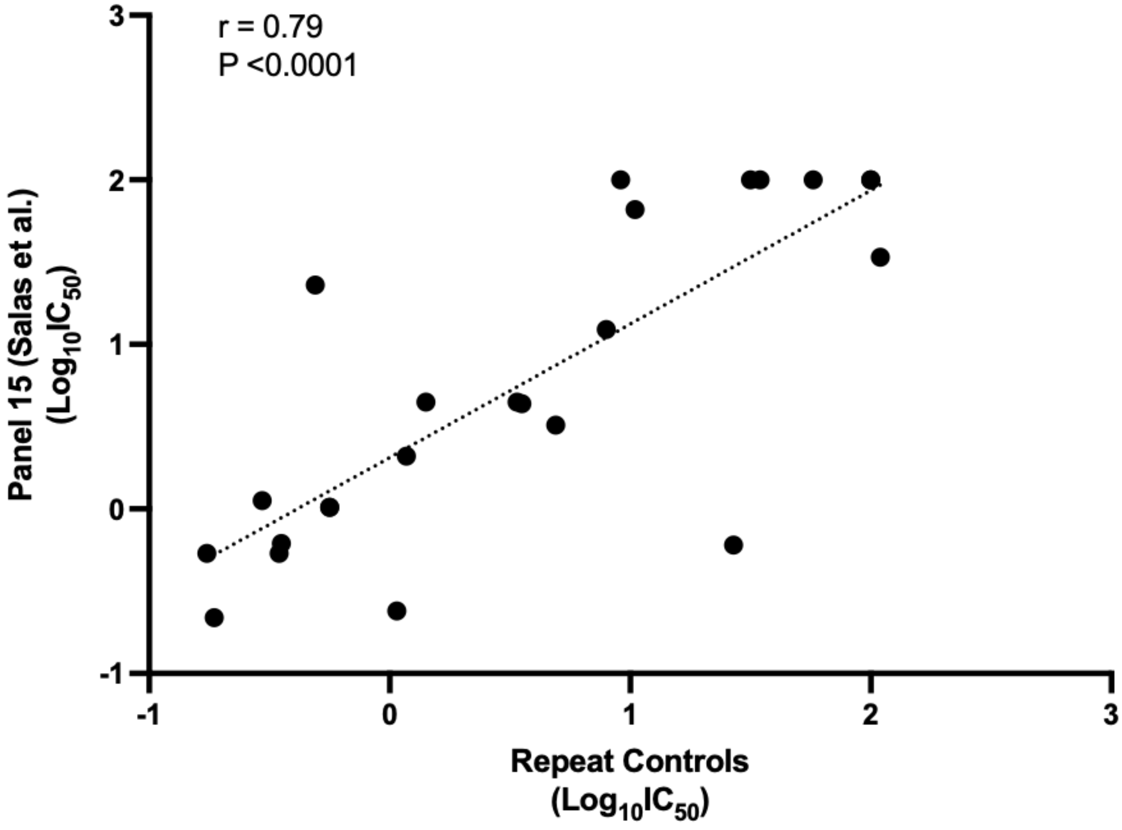

Supplementary Figure 1. Independent measurements of neutralization of the same HCVpp in the current study and results obtained previously by Salas et al. (1) were significantly correlated. To confirm reproducibility, neutralization of four HCVpp from the published panel of 15 HCVpp (UKNP1.11.6, 1a154, 1a72 and UKNP1.18.1) were re-tested in parallel with the 12 new gt 2-6 HCVpp, using seven neutralizing antibodies. Each point indicates neutralization of one HCVpp by one mAb in this study and in Salas et al. The mAbs HCV1 and AP33 bind highly similar epitopes so results with these two mAbs were averaged for each HCVpp. R and p-values obtained using Pearson’s correlation.

mAb	Tier	E1E2 Variant	412	413	414	415	416	417	418	419	420	421	422	423	IC50 (ug/mL)	# subs
HCV1	2	JHP2a.2	Q	L	I	N	T	N	G	S	W	H	I	N	0.17	0
	1	JHP2l.1	Q	L	I	N	T	N	G	S	W	H	I	N	1.01	0
	2	JHP3i.1	Q	L	I	N	T	N	G	S	W	H	I	N	1.68	0
	3	JHP6e.1	Q	L	V	N	T	N	G	S	W	H	I	N	0.40	1
	3	JHP3a.C48d84	Q	L	V	N	T	N	G	S	W	H	I	N	1.45	1
	3	JHP3h.1	Q	L	I	N	S	N	G	S	W	H	I	N	2.29	1
	3	JHP2m.1	Q	L	V	N	T	N	G	S	W	H	I	N	4.32	1
	2	JHP5.2	Q	L	I	N	S	N	G	S	W	H	I	N	4.57	1
	3	JHP5.1	Q	L	I	N	S	N	G	S	W	H	I	N	11.30	1
	4	JHP2j.1	Q	L	I	N	T	N	G	S	W	H	V	N	31.26	1
	3	JHP4.1	Q	L	V	N	S	N	G	S	W	H	I	N	5.55	2
	2	JHP2b.2	N	L	I	K	T	N	G	S	W	H	I	N	100.00	2

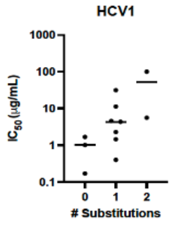

mAb	Tier	E1E2 Variant	412	413	414	415	416	417	418	419	420	421	422	423	IC50 (ug/mL)	# subs
AP33	2	JHP2a.2	Q	L	I	N	T	N	G	S	W	H	I	N	0.08	0
	2	JHP3i.1	Q	L	I	N	T	N	G	S	W	H	I	N	1.10	0
	4	JHP2j.1	Q	L	I	N	T	N	G	S	W	H	I	N	39.23	0
	2	JHP5.2	Q	L	I	N	S	N	G	S	W	H	I	N	0.11	1
	1	JHP2l.1	Q	L	I	N	T	N	G	S	W	H	V	N	0.70	1
	3	JHP6e.1	Q	L	V	N	T	N	G	S	W	H	I	N	1.79	1
	3	JHP3h.1	Q	L	I	N	S	N	G	S	W	H	I	N	3.51	1
	3	JHP3a.C48d84	Q	L	V	N	T	N	G	S	W	H	I	N	5.07	1
	3	JHP2m.1	Q	L	V	N	T	N	G	S	W	H	I	N	9.17	1
	3	JHP5.1	Q	L	I	N	S	N	G	S	W	H	I	N	11.20	1
	3	JHP4.1	Q	L	V	N	S	N	G	S	W	H	I	N	4.01	2
	2	JHP2b.2	N	L	I	K	T	N	G	S	W	H	I	N	100.00	2

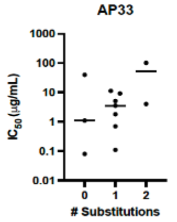

mAb	Tier	E1E2 Variant	408	409	410	411	412	413	414	415	416	417	418	419	420	421	422	423	IC50 (ug/mL)	# subs
H33.4	2	JHP5.2	R	Q	N	L	Q	L	I	N	S	N	G	S	W	H	I	N	0.11	0
	2	JHP3i.1	R	Q	N	L	Q	L	I	N	T	N	G	S	W	H	I	N	16.49	1
	3	JHP5.1	K	Q	N	I	Q	L	I	N	S	N	G	S	W	H	I	N	6.99	2
	3	JHP4.1	Q	Q	N	L	Q	L	V	N	S	N	G	S	W	H	I	N	100.00	2
	3	JHP3h.1	Q	Q	N	I	Q	L	I	N	S	N	G	S	W	H	I	N	100.00	2
	1	JHP2l.1	K	Q	N	I	Q	L	I	N	T	N	G	S	W	H	I	N	3.20	2
	3	JHP6e.1	Q	Q	N	L	Q	L	V	N	T	N	G	S	W	H	I	N	49.56	3
	2	JHP2a.2	Q	Q	N	I	Q	L	I	N	T	N	G	S	W	H	I	N	100.00	3
	3	JHP3a.C48d84	N	Q	N	L	Q	L	V	N	T	N	G	S	W	H	I	N	100.00	3
	3	JHP2m.1	Q	Q	K	L	Q	L	V	N	T	N	G	S	W	H	I	N	41.31	4
	4	JHP2j.1	S	Q	N	V	Q	L	I	N	T	N	G	S	W	H	V	N	100.00	4
	2	JHP2b.2	Q	Q	K	L	N	L	I	K	T	N	G	S	W	H	I	N	7.81	5

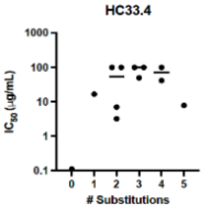

mAb	Tier	E1E2 Variant	421	441	442	443	446	529	613	IC50 (ug/mL)	# subs
HC84.26	2	JHP2a.2	H	L	F	Y	R	W	Y	0.07	0
	3	JHP4.1	H	L	F	Y	R	W	Y	0.61	0
	1	JHP2l.1	H	L	F	Y	S	W	Y	0.09	1
	3	JHP3a.C48d84	H	L	F	Y	K	W	Y	0.11	1
	3	JHP6e.1	H	L	F	Y	K	W	Y	0.15	1
	2	JHP2b.2	H	L	F	Y	K	W	Y	0.52	1
	3	JHP5.1	H	L	F	Y	K	W	Y	0.69	1
	3	JHP3h.1	H	L	F	Y	K	W	Y	1.08	1
	3	JHP2m.1	H	L	F	Y	S	W	Y	9.10	1
	4	JHP2j.1	H	L	F	Y	N	W	Y	20.69	1
	2	JHP5.2	H	L	M	Y	N	W	Y	0.35	2
	2	JHP3i.1	H	L	M	Y	K	F	Y	2.45	3

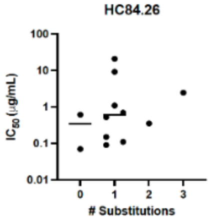

mAb	Tier	E1E2 Variant	427	428	429	430	431	438	439	442	443	529	531	IC50 (ug/mL)	# subs
AR3A	1	JHP2l.1	L	N	C	N	D	I	T	F	Y	W	A	0.02	0
	2	JHP2b.2	L	N	C	N	D	I	A	F	Y	W	E	0.72	2
	3	JHP6e.1	L	N	C	N	D	I	A	F	Y	W	E	1.65	2
	3	JHP5.1	L	N	C	N	D	I	A	F	Y	W	E	4.16	2
	3	JHP3a.C48d84	L	N	C	N	E	I	A	F	Y	W	A	10.25	2
	3	JHP2m.1	L	N	C	N	D	I	A	F	Y	W	E	13.14	2
	2	JHP5.2	L	N	C	N	D	I	A	M	Y	W	S	0.18	3
	2	JHP2a.2	L	N	C	N	D	L	A	F	Y	W	E	0.50	3
	3	JHP4.1	L	N	C	N	D	L	A	F	Y	W	E	5.10	3
	3	JHP3h.1	L	N	C	N	D	L	A	F	Y	W	E	8.08	3
	4	JHP2j.1	L	N	C	N	D	L	A	F	Y	W	E	14.28	3
	2	JHP3i.1	L	N	C	N	D	I	A	M	Y	F	E	2.26	4

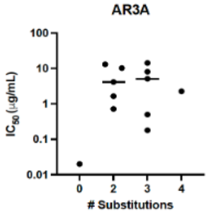

mAb	Tier	E1E2 Variant	421	515	523	546	635	637	639	IC50 (ug/mL)	# subs
CBH-7	1	JHP2l.1	H	V	G	S	G	E	R	4.22	0
	3	JHP3a.C48d84	H	V	G	S	G	E	R	100.00	0
	2	JHP2b.2	H	V	G	Q	G	E	R	15.32	1
	2	JHP5.2	H	V	G	T	G	E	R	30.98	1
	2	JHP2a.2	H	V	G	Q	G	E	R	100.00	1
	2	JHP3i.1	H	V	G	Q	G	E	R	100.00	1
	3	JHP4.1	H	V	G	R	G	E	R	100.00	1
	3	JHP6e.1	H	V	G	K	G	E	R	100.00	1
	3	JHP5.1	H	V	G	R	G	E	R	100.00	1
	3	JHP3h.1	H	V	G	A	G	E	R	100.00	1
	3	JHP2m.1	H	V	G	Q	G	E	R	100.00	1
	4	JHP2j.1	H	V	G	R	G	E	R	100.00	1

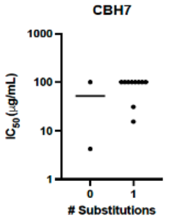

mAb	Tier	E1E2 Variant	649	667	696	698	IC50 (ug/mL)	# subs
AR4A	2	JHP2a.2	G	H	I	D	0.18	0
	2	JHP5.2	G	H	I	D	0.47	0
	2	JHP3i.1	G	H	I	D	0.68	0
	3	JHP3a.C48d84	G	H	I	D	0.69	0
	2	JHP2b.2	G	H	I	D	0.72	0
	1	JHP2l.1	G	H	I	D	1.42	0
	3	JHP5.1	G	H	I	D	4.22	0
	3	JHP3h.1	G	H	I	D	4.45	0
	3	JHP2m.1	G	H	I	D	16.13	0
	3	JHP4.1	G	L	I	D	1.31	1
	3	JHP6e.1	G	F	I	D	1.64	1
	4	JHP2j.1	G	H	T	D	100.00	1

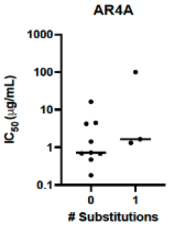

Supplementary Figure 2. Association of amino acid polymorphisms in the mAb binding epitopes of E1E2 variants with neutralization resistance. For each mAb, the most sensitive E1E2 variant determined by the lowest IC_{50} is listed first and highlighted in green. Amino acid polymorphisms relative to the most sensitive variant are denoted in red. Epitopes were defined based on CryoEM or x-ray crystal structures of mAb-epitope complexes, as well as available site-directed mutagenesis data as follows: HC33.4 (aa 408-423) (2, 3), AP33 (4, 5) and HCV1 (6) (aa 412-423), AR4A (aa 649, 667, 696, 698) (7), HC84.26 (aa 421,441-443, 446, 529, 613), CBH7 (aa 421, 515, 523, 546, 635, 637, 639) (8), and AR3A (aa 427-431, 438-439, 442-443, 529, 531) (9). The epitope residues for HC84.26 were identified using an affinity matured form of HC84.26, HC84.26AD. Amino acid numbering is relative to strain H77. Graphs show the number of epitope substitutions of each variant relative to the most sensitive variant for that mAb. Horizontal lines are medians. Groups were compared by Kruskal-Wallis test with Dunn's adjustment for multiple comparisons. None of the differences between groups were statistically significant.

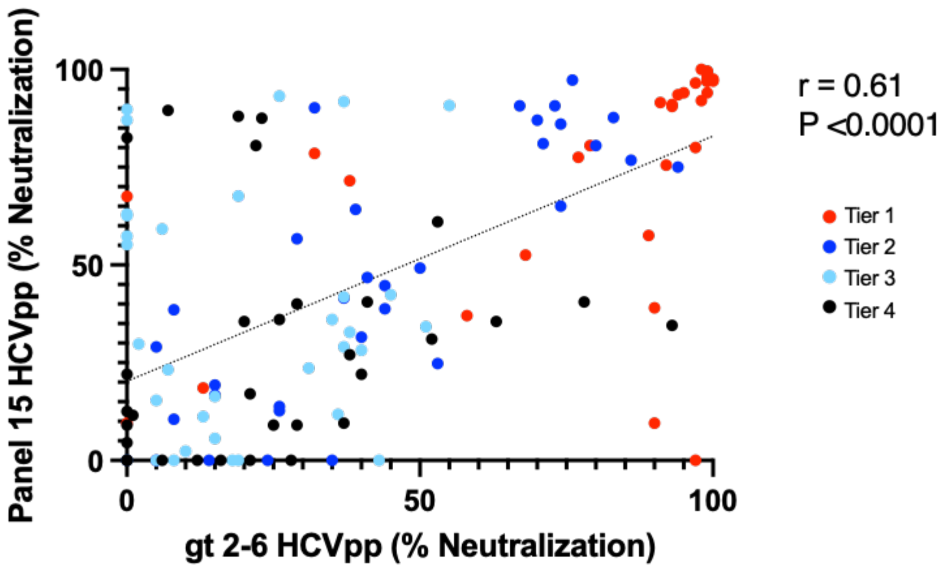

Supplementary Figure 3. Plasma neutralization measured using the panel of 15 HCVpp or the gt 2-6 HCVpp panel are significantly correlated. Percent neutralization of 33 plasma samples from gt 1-6-infected persons were measured using the panel of 15 HCVpp in Salas et al. (1) or gt 2-6 HCVpp in this study. Each point is the average percent neutralization of one tier of each HCVpp panel by each plasma sample. Neutralization values <0 were normalized to 0. Two plasma samples (subject 22 and 30) from Salas et al. were excluded in this analysis due to unavailability of samples. Tiers are indicated by color. R and p-values are obtained using Pearson's correlation.

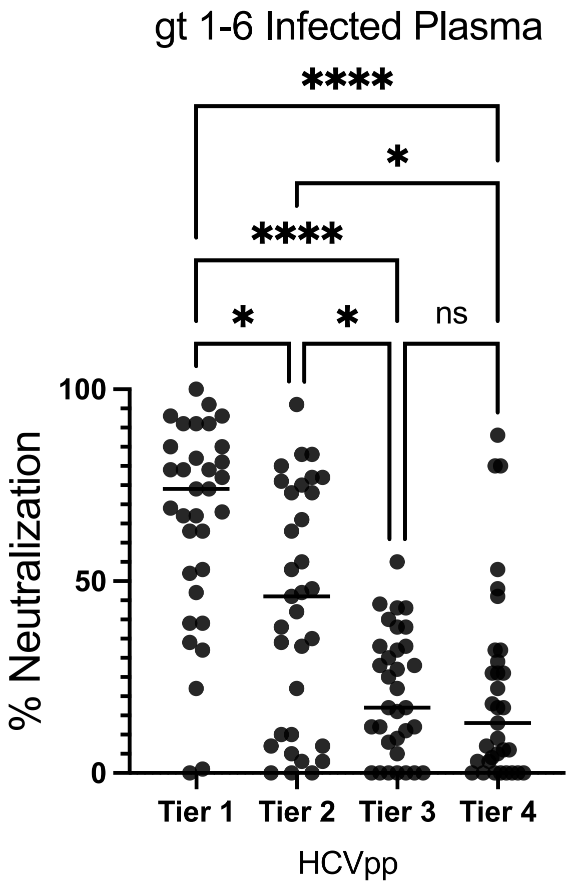

Supplementary Figure 4. Neutralization by plasma from gt 1-6 infected persons using Tier 1-4 variants from the gt 2-6 HCVpp panel. Percent neutralization of 33 plasma samples at 1:20 dilution from gt 1-6-infected persons were measured with the gt 2-6 HCVpp panel. Each point indicates the percent neutralization of a single HCVpp by a single plasma sample. Neutralization values less than zero were normalized to zero. Horizontal lines indicate medians. Groups were compared by the Kruskal Wallis test with Dunn's adjustment for multiple comparisons. ns, not significant; * = $P < .05$, **** = $P < 0.0001$.

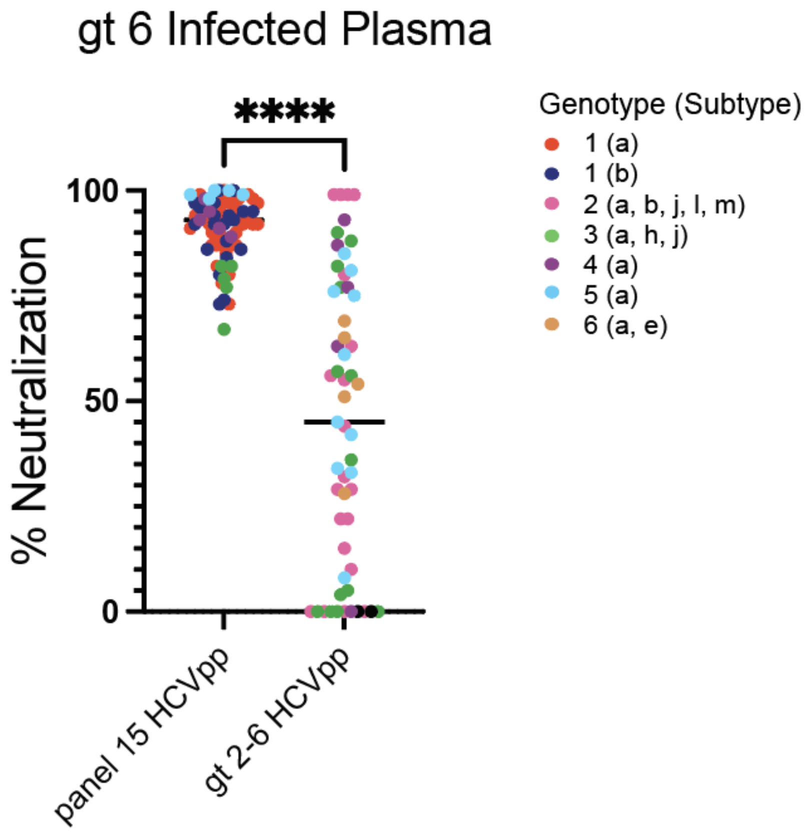

Supplementary Figure 5. The panel of gt 2-6 HCVpp was more resistant to neutralization by plasma from gt 6-infected persons, relative to the panel of 15 HCVpp. Percent neutralization of 5 plasma samples at 1:20 dilution from genotype 6-infected persons were measured with the panels of 15 HCVpp in Salas et al. (1) and gt 2-6 HCVpp in this study. Each point indicates the percent neutralization of a single HCVpp by a single plasma sample. Neutralization values <0 were normalized to zero. Groups were non-normally distributed and were compared by Kolmogorov-Smirnov's test. **** = $P < 0.0001$.

Supplementary Materials References

1. Salas JH, Urbanowicz RA, Guest JD, Frumento N, Figueroa A, Clark KE, Keck Z, Cowton VM, Cole SJ, Patel AH, Fuerst TR, Drummer HE, Major M, Tarr AW, Ball JK, Law M, Pierce BG, Foung SKH, Bailey JR. 2022. An Antigenically Diverse, Representative Panel of Envelope Glycoproteins for Hepatitis C Virus Vaccine Development. Gastroenterology 162:562-574.
2. Keck Z, Wang W, Wang Y, Lau P, Carlsen TH, Prentoe J, Xia J, Patel AH, Bukh J, Foung SK. 2013. Cooperativity in virus neutralization by human monoclonal antibodies to two adjacent regions located at the amino terminus of hepatitis C virus E2 glycoprotein. JVirol 87:37-51.
3. El-Diwany R, Cohen VJ, Mankowski MC, Wasilewski LN, Brady JK, Snider AE, Osburn WO, Murrell B, Ray SC, Bailey JR. 2017. Extra-epitopic hepatitis C virus polymorphisms confer resistance to broadly neutralizing antibodies by modulating binding to scavenger receptor B1. PLoS Pathog 13:e1006235.
4. Pantua H, Diao J, Ultsch M, Hazen M, Mathieu M, McCutcheon K, Takeda K, Date S, Cheung TK, Phung Q, Hass P, Arnott D, Hongo JA, Matthews DJ, Brown A, Patel AH, Kelley RF, Eigenbrot C, Kapadia SB. 2013. Glycan shifting on hepatitis C virus (HCV) E2 glycoprotein is a mechanism for escape from broadly neutralizing antibodies. J Mol Biol 425:1899-1914.
5. Owsianka A, Tarr AW, Juttla VS, Lavillette D, Bartosch B, Cosset FL, Ball JK, Patel AH. 2005. Monoclonal antibody AP33 defines a broadly neutralizing epitope on the hepatitis C virus E2 envelope glycoprotein. JVirol 79:11095-11104.
6. Broering TJ, Garrity KA, Boatright NK, Sloan SE, Sandor F, Thomas WD, Jr., Szabo G, Finberg RW, Ambrosino DM, Babcock GJ. 2009. Identification and characterization of broadly neutralizing human monoclonal antibodies directed against the E2 envelope glycoprotein of hepatitis C virus. JVirol 83:12473-12482.
7. de la Peña AT, Sliepen K, Eshun-Wilson L, Newby M, Allen JD, Koekkoek S, Zon I, Chumbe A, Crispin M, Schinkel J, Lander GC, Sanders RW, Ward AB. 2021. Structure of the hepatitis C virus E1E2 glycoprotein complex. bioRxiv doi:10.1101/2021.12.16.472992:2021.12.16.472992.
8. Shahid S, Karade SS, Hasan SS, Yin R, Jiang L, Liu Y, Felbinger N, Kulakova L, Toth EA, Keck ZY, Foung SKH, Fuerst TR, Pierce BG, Mariuzza RA. 2025. Cryo-EM structures of HCV E2 glycoprotein bound to neutralizing and non-neutralizing antibodies determined using bivalent Fabs as fiducial markers. Commun Biol 8:825.
9. Tzarum N, Giang E, Kong L, He L, Prentoe J, Augestad E, Hua Y, Castillo S, Lauer GM, Bukh J, Zhu J, Wilson IA, Law M. 2019. Genetic and structural insights into broad neutralization of hepatitis C virus by human VH1-69 antibodies. Sci Adv 5:eaav1882.
